# Outcomes of patients with sarcoma enrolled in clinical trials of pazopanib combined with histone deacetylase, mTOR, Her2, or MEK inhibitors

**DOI:** 10.1038/s41598-017-13114-8

**Published:** 2017-11-21

**Authors:** Vikas Dembla, Roman Groisberg, Ken Hess, Siqing Fu, Jennifer Wheler, David S. Hong, Filip Janku, Ralph Zinner, Sarina Anne Piha-Paul, Vinod Ravi, Robert S. Benjamin, Shreyaskumar Patel, Neeta Somaiah, Cynthia E. Herzog, Daniel D. Karp, Jason Roszik, Funda Meric-Bernstam, Vivek Subbiah

**Affiliations:** 1 Department of Investigational Cancer Therapeutics (Phase 1 Program), Division of Cancer Medicine, The University of Texas MD Anderson Cancer Center, Houston, TX USA; 2 Department of Sarcoma Medical Oncology, Division of Cancer Medicine, The University of Texas MD Anderson Cancer Center, Houston, TX USA; 3 Department of Biostatistics,The University of Texas MD Anderson Cancer Center, Houston, TX USA; 4 Novartis, Cambridge, MA USA; 5 Thomas Jefferson Hospital, Philadelphia, PA United States; 6 Division of Pediatrics,The University of Texas MD Anderson Cancer Center, Houston, Texas United States; 7 Department of Melanoma Medical Oncology,The University of Texas MD Anderson Cancer Center, Houston, Texas United States; 8 Department of Genomic Medicine, The University of Texas MD Anderson Cancer Center, Houston, Texas United States

## Abstract

Pazopanib is US FDA approved for the treatment of advanced soft tissue sarcomas. All patients with this disease ultimately develop resistance to therapy. Mechanisms of resistance include activation of the mTOR, histone deacetylase (HDAC), MAPK, and ERBB4 pathways. We hypothesized that combining pazopanib with other targeted agents inhibiting these pathways would increase response rates. We retrospectively evaluated the safety and efficacy of pazopanib plus vorinostat, everolimus, lapatinib or trastuzumab, and MEK inhibitor in patients with advanced sarcoma. The Cancer Geneome Atlas (TCGA) data was analyzed for HDAC, PI3K, HER2, and MAPK/RAS/RAF gene alterations from sarcoma TCGA. Of the 44 advanced sarcoma patients in these trials, 27 (61%) were male; 18 (41%) had bone sarcoma, and 26 (59%) had soft tissue sarcoma. Best response was partial response (PR) in four patients [(overall response rate **(**ORR) = 9%, 95% confidence interval [CI] 3% to 22%)]. The median progression-free survival (PFS) for all patients was 9.6 weeks (95% CI 8.0 to 15.7 weeks). Analysis of TCGA data revealed HDAC, PI3K, HER2, and MAPK/RAS/RAF gene alterations in 112/243 (46%) of patients predominantly HDAC1–11 (41%) alterations. Pazopanib combinations did demonstrate safety in combination with other agents. TCGA data suggests further evaluation of epigenetic pathway inhibitors in sarcoma.

## Introduction

Sarcomas are rare mesenchymal neoplasms with over 50 different subtypes. Chemotherapy-based treatment algorithms have been the mainstay for sarcomas other than gastrointestinal stromal tumors. Pazopanib, a multi-kinase vascular endothelial growth factor (VEGF) based tyrosine kinase inhibitor (TKI) was the first targeted therapy approved in 2012 in the United States for the treatment of patients with advanced and metastatic soft tissue sarcomas who have progressed on standard chemotherapy (anthracycline as well as gemcitabine or ifosfamide). Pazopanib’s approval was based on the results of the PALETTE study, a randomized phase 3 study done in 72 institutions across 13 countries in which 369 patients were randomized in a 2:1 fashion to receive either pazopanib at 800 mg daily dose or placebo, with no crossover allowed after progression^1^. The primary end point was progression-free survival (PFS). The study was able to meet its primary end point, as pazopanib increased PFS by 3 months over placebo (4.6 months vs 1.6 months, hazard ratio [HR] = 0.31, 95% confidence interval [CI] 0.24 to 0.40; p < 0.0001). Ninety-three percent of patients had received prior anthracycline-based chemotherapy. There was a trend towards an increase in overall survival (OS) with pazopanib, but the increase was not statistically significant (p = 0.25).

Most patients with sarcoma who are on pazopanib ultimately develop resistance to it, leading to progression of disease, and a major challenge in the treatment of advanced soft tissue sarcoma remains a lack of predictive biomarkers to guide further therapy^2^. In addition, attempts to combine pazopanib with chemotherapy has been quite challenging, as the combination was associated with toxicity and did not improve upon the response of either agent^3^. The mechanisms of resistance to multi-kinase anti–vascular endothelial growth factor (VEGF) drugs such as pazopanib are complex and diverse. These mechanisms may be intrinsic or acquired^4^. Mechanisms of primary resistance to anti-VEGF drugs include activation of alternative receptor tyrosine kinases such as the mechanistic target of rapamycin (mTOR), histone deacetylase (HDAC), mitogen-activated protein kinase (MAPK), and ERBB4 pathways^5^. A previous trial demonstrated activity of pazopanib with the mTOR inhibitor everolimus against refractory solid tumors^6^. We hypothesized that combining pazopanib with inhibitors of pathways involved in resistance to anti-VEGF drugs would increase response rates and overcome resistance to prior therapy with pazopanib in patients with sarcoma.

We therefore retrospectively evaluated the safety and efficacy of pazopanib combined with an inhibitor of HDAC, mTOR, Her2, or MEK in patients with advanced and refractory sarcoma enrolled in phase 1 trials of these combinations. We also analyzed the Cancer Genome Atlas (TCGA) data for these respective pathway alterations.

## Patients and Methods

### Patient Selection and Treatment

We reviewed records of sarcoma patients enrolled in clinical trials of pazopanib combinations. Patients with advanced, refractory, and/or metastatic sarcoma were selected for our analysis. The trials had been individually approved by the Institutional Review Board and conducted at The University of Texas MD Anderson Cancer Center in accordance with Institutional Review Board guidelines. The retrospective review was approved by the Institutional Review Board as well.

Medical records were retrospectively searched for patients enrolled in the phase 1 trials of pazopanib plus vorinostat (HDAC inhibitor; NCT01339871)^7^, pazopanib plus everolimus (mTOR inhibitor; NCT01430572)^6^, pazopanib plus lapatinib or trastuzumab (Her2 inhibitor; NCT01454804), and pazopanib plus a MEK inhibitor (NCT01438554). All patients included in the trials were 14 years of age or older; had histologically confirmed, measurable or evaluable advanced sarcoma that had progressed before study entry; and an Eastern Cooperative Oncology Group (ECOG) performance status of 0 to 2^8^. The patients were also required to have adequate marrow function, serum creatinine level ≤2 times the upper limit of normal, total bilirubin level of ≤2.0 mg/dL, alanine and aspartate aminotransferase level ≤2.5 times the upper limit of normal or ≤5 times the upper limit of normal if liver metastases were present. Excluded from the trials were patients with poorly controlled hypertension, clinically significant cardiovascular disease, symptomatic involvement of their cancer in the central nervous system, and other comorbidities; patients who were pregnant or lactating; and patients unwilling or unable to provide written informed consent. Details of the eligibility criteria depended on the respective protocols (NCT01339871, NCT01430572, NCT01454804, and NCT01438554).

In selecting patients for the retrospective review, we excluded patients who consented but never started the treatment owing to financial or insurance reasons, who elected to go to hospice, or who had disease progression before the combination drugs could be given.

Treatment was administered per the recommended doses shown in Table 1. The duration of each cycle varied depending on the combination regimen used. Treatment was continued as long as the patient had no evidence of tumor progression or prohibitive toxicity. Molecular correlates of response were analyzed when available.

**Table 1:** PFS and dosage by pazopanib combinations in the Pazo + study.

Drug combined with pazopanib	Number of patients	Median PFS	Dosage of experimental agent + dosage of pazopanib (# of pts)
Vorinostat	22	8.4 weeks	100 mg daily (n = 3)
			200 mg daily (n = 9)
			300 mg daily (n = 9)
			400 mg daily (n = 1)
Trametinib	13	13.7 weeks	2 mg + 800 mg daily (n = 10)
			2 mg + 600 mg daily (n = 2)
			1 mg + 800 mg daily (n = 1)
Everolimus	6	9.9 weeks	5 mg + 400 mg daily (n = 2)
			7.5 mg + 400 mg daily (n = 2)
			10 mg + 600 mg daily (n = 2)
Lapatinib	2	12.0 weeks	750 mg daily + 400 mg every other day (n = 2)
Trastuzumab	1	18.0 weeks	Loading dose 4 mg/kg followed by maintenance dose 2 mg/kg + 400 mg daily (n = 1)

### Safety and Efficacy Evaluation

All patients who received at least one dose on study were considered evaluable for the safety and efficacy of the agents. We extracted the following information from the patients’ medical records for our analysis: histologic diagnosis (all histologic findings had been centrally reviewed at MD Anderson Cancer Center), tumor associated mutations or aberrations, entry date for the trial, date of progression of disease, date of last follow-up at the phase 1 clinic or date of death, ECOG performance status at enrollment, any prior treatment with pazopanib, any dose-limiting toxicity, and any other grade 3 or 4 adverse events within the first 28 days of treatment per Common Terminology Criteria for Adverse Events (CTCAE) v4.0. Dose-limiting toxicity was defined as any treatment-related adverse event leading to dose modification after cycle 1, including grade 4 prolonged hematologic toxicity, grade 4 prolonged nausea or vomiting, or grade 4 fatigue or hypertension, as described previously. The Royal Marsden Hospital (RMH) prognostic score was calculated using serum lactate dehydrogenase, serum albumin values, and the number of metastatic sites involved. Radiographic imaging studies were performed approximately every two cycles (every 8 weeks) of therapy. The response rate was assessed according to RECIST version 1.1^9^.

### Statistical Methods

Categorical variables were summarized using frequencies and relative frequencies. Continuous variables are summarized using median and range. PFS was defined as the time from cycle 1 day 1 to the date of progression or death, whichever came first. Patients who were alive and progression free at the last clinical follow-up were censored at the date of the last clinical follow-up. OS was defined as the time from cycle 1 day 1 to death from any cause. Patients alive at last contact were censored at the date of last contact. OS and PFS distributions were estimated using the Kaplan-Meier method. Hazard ratios and corresponding confidence intervals and p values were computed using Cox proportional hazards regression analysis. Clopper-Pearson exact binomial confidence intervals were provided for estimates of proportions.

### Analysis of The Cancer Genome Atlas (TCGA)

We analyzed next generation sequencing data from the Cancer Genome Atlas (TCGA). Figures were generated to show copy number alterations, and mutations in selected genes namely HDAC, PI3K, HER2, and MAPK/RAS/RAF. Figure panel was created using the cBioPortal [ref: https://www.ncbi.nlm.nih.gov/pubmed/22588877] for the sarcoma data set available on the portal. In addition we assessed associations between HDAC alterations and other alterations (i.e., is the rate of HDAC alterations higher in patients with the other alteration compared to patients without those alterations). The McNemar’s chi-squared test for paired proportions was used for statistical analysis.

## Results

Forty-four patients with sarcoma were identified who were enrolled on phase 1 trials of pazopanib combinations. Patient characteristics are shown in Table 2. The HDAC inhibitor vorinostat (Zolinza®) was given to 22 patients (50%), the Her2 inhibitor lapatinib (Tykerb®) or the Her2 inhibitor trastuzumab (Herceptin®) was given to three patients (7%), the mTOR inhibitor everolimus (Afinitor®) was given to six patients (14%), and the MEK inhibitor trametinib (Mekinist®) was given to 13 patients (30%) (Table 2). Demographic analysis revealed 27 male (61%) and 17 female (39%) patients with a median age of 32.5 years and an age range of 14 to 77 years. Patients with bone sarcomas (Ewing sarcoma, osteosarcoma, and chondrosarcoma) constituted 41% of our cohort (18 patients). The other 59% (26 patients) had 13 different types of soft tissue sarcomas including leiomyosarcoma, alveolar soft part sarcoma, liposarcoma (DDLS and WDLS), and others. Trial entry dates ranged from June 2011 to July 2015. Most patients had an ECOG performance status of 1 (35 patients), indicating restriction in physically strenuous activity only. Prior kinase inhibitor use was permitted on these trials and twelve patients had previously received a targeted therapy. One ASPS patient had previously been treated with cedirinib. One hemangiopericytoma received sunitinib. An ASPS and an unclassified high grade sarcoma each had prior MET inhibitor. Three patients each were treated with temsirolimus or MDM2 inhibitior. One patient was pre-treated with sorafenib and another with a PI3K inhibitor.

**Table 2:** Patient characteristics of patients enrolled in pazopanib based trials (n = 44)

Characteristic	Value
Sex, No.	
Male	27
Female	17
Age	
Range	14–77 years
Median	32.5 years
ECOG performance status, No.	
0	7
1	35
2	2
Bone sarcoma, No. (%)	
Ewing	11 (25%)
Osteosarcoma	5 (11%)
Chondrosarcoma	2 (5%)
Soft tissue sarcoma, No. (%)	
Leiomyosarcoma	6 (14%)
Alveolar soft part sarcoma	3 (7%)
Liposarcoma	2 (5%)
Others	15 (34%)
Prior pazopanib, No.	
Yes	5
No	39
No. of metastatic sites, No.	
0	1
1	20
2	17
3	4
4	2

ECOG: Eastern Cooperative Oncology Group.

### Responses, PFS and OS

**Figure 1:**
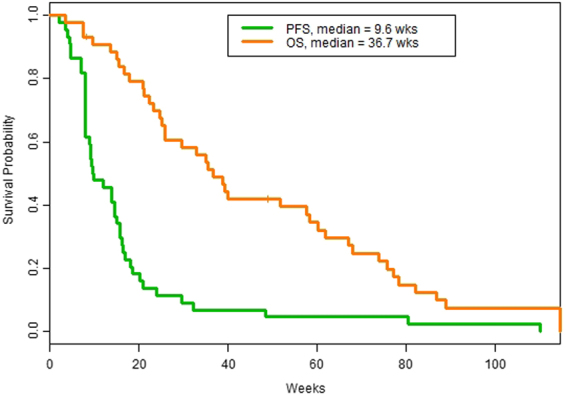
Kaplan-Meier curves for PFS and OS.

**Figure 2:**
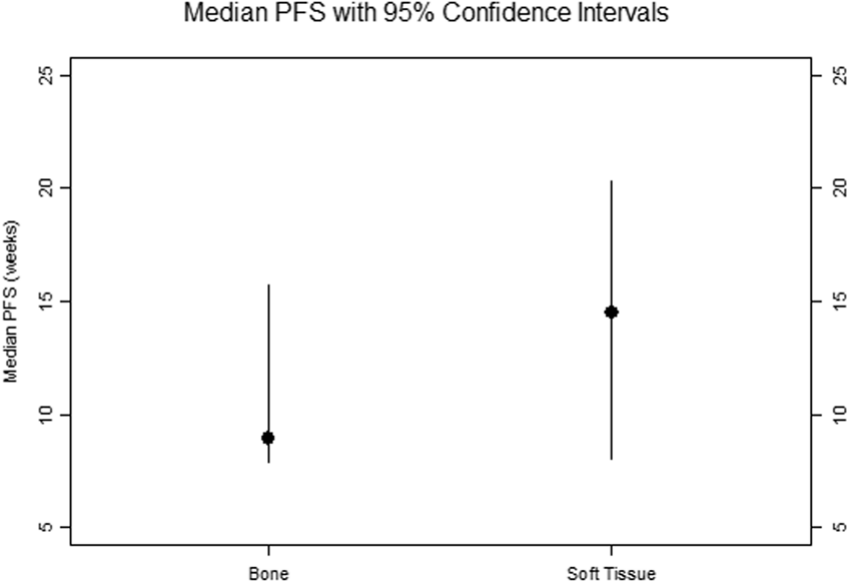
PFS outcomes for patients with sarcoma.

**Figure 3:**
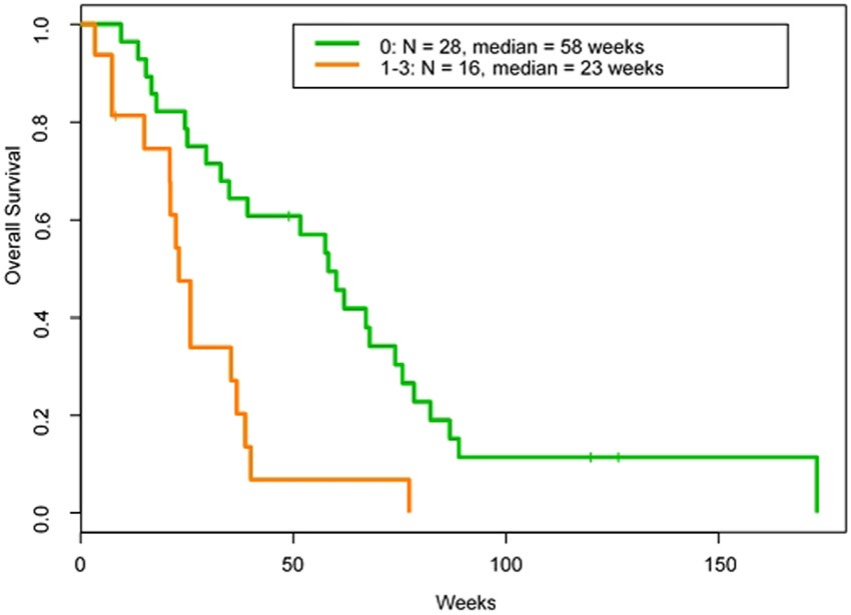
OS by RMH prognostic score.

**Figure 4:**
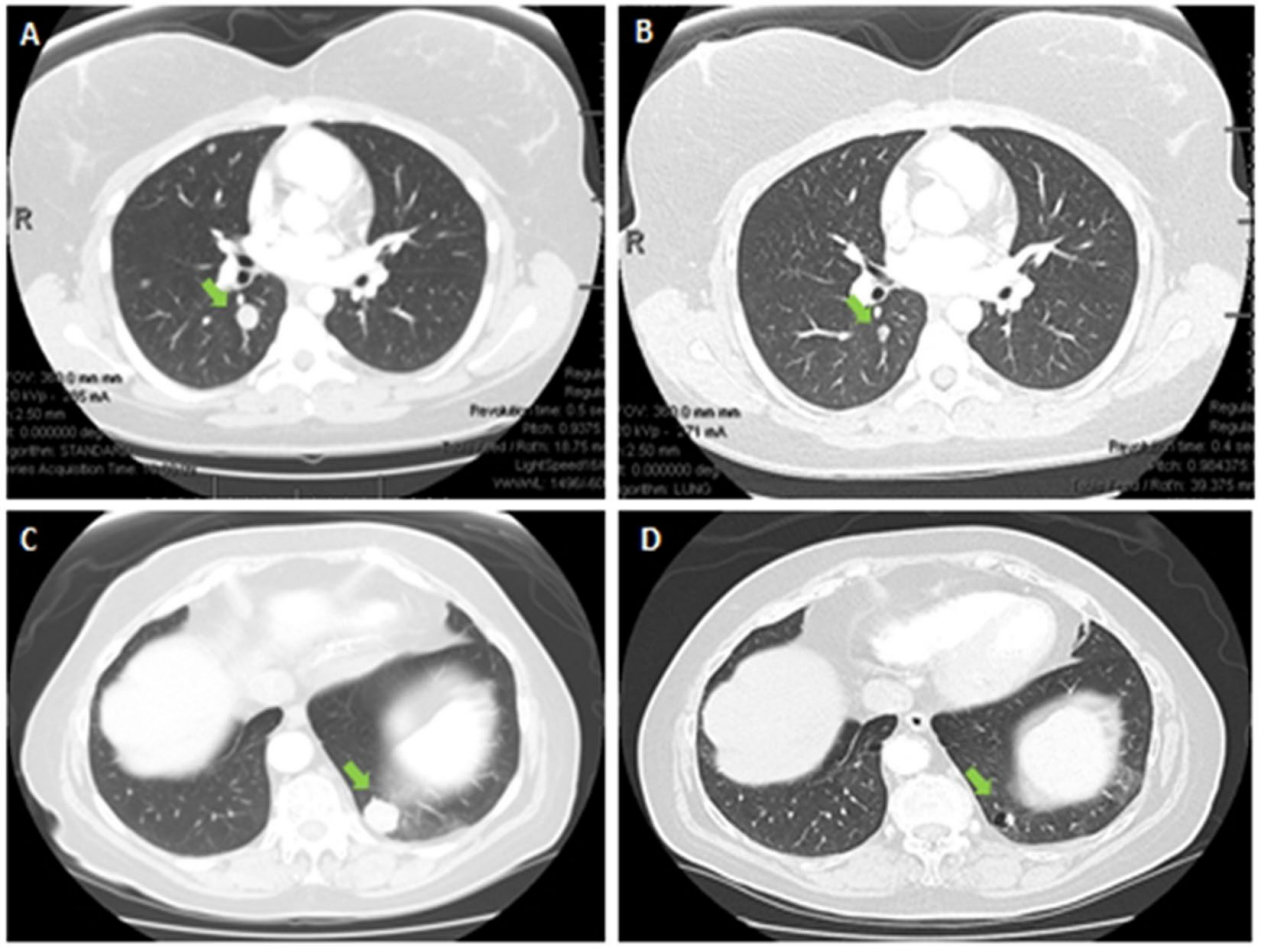
Pre- and post-treatment computed tomography scans of patients with sarcoma showing partial responses to pazopanib combinations. (**A**), (**B**) Alveolar soft part sarcoma before (**A**) and after (**B**) treatment, with 40% decrease in size of lung metastases (green arrow) per RECIST. (**C**,**D)** Angiosarcoma of the scalp metastatic to the lung before (**C**) and after (**D**) treatment, with 54% reduction in size of lung metastases per RECIST.

The median PFS for all patients was 9.6 weeks (95% CI 8.0 to 15.7 weeks) as shown in Table 1, Fig. 1 and Fig. 2. The median OS for all patients was 36.7 weeks (95% CI 25.9 to 60.1), as shown in Fig. 3. The median PFS was 14.5 weeks for soft tissue sarcoma patients and 8.9 weeks for bone sarcoma patients (hazard ratio [HR] = 0.5, 95% CI 0.3 to 1.0, p = 0.038. The median OS was 35.2 weeks for bone sarcoma patients and 38.7 weeks for soft tissue sarcoma patients (HR = 0.8, 95% CI 0.4 to 1.5, p = 0.43). There were 43 patients with evaluable response data, 1 patient in the vorinostat arm was intolerant to treatment and could not undergo restaging scans. Clinical benefit rate from combination treatment defined as the total number of complete responses, partial responses, and stable disease at >6 months, occurred in eight of the 43 evaluable patients (19%; 95% CI 8% to 33%. Sixteen patients had stable disease, whereas 23 patients had progression of disease. The best responses were partial responses in four patients (ORR = 9%, 95% CI 3% to 22%) as shown in Table 3. Figures 4A and 4B show the computed tomography scans of a patient who had a 40% decrease in size of lung metastases per RECIST, and Fig. 4C and D are those of a patient who had a 54% decrease in size of lung metastases per RECIST. There were no complete responses. Most patients (39/44, 89%) had not been pretreated with pazopanib. Only five patients had previously received pazopanib as a single agent, and all five had progressive disease on the combination treatment in our study.

**Table 3:** Responses in pazopanib combination trials.

Best response obtained	Number of patients
Stable disease	16
Progression of disease	23
Partial response	4
Intolerant to treatment	1

### Doses of pazopanib

Doses of pazopanib ranged from 400 mg to 800 mg. Five of the 16 patients who had a starting dose of 800 mg had to have their dose reduced owing to adverse events. These dose reductions were not seen across any one trial; four of the patients with dose reductions were in the vorinostat cohort, and one was in the MEK inhibitor cohort. One patient had to have a dose reduction from a starting dose of 600 mg, and one patient went off the study owing to drug intolerance at 600 mg. No dose reductions were needed with a 400-mg starting dose. Dose reductions were required independent of the partner drug.

Twelve of 44 patients experienced dose-limiting toxicity (27%, 95% CI 15% to 43%). Grade 3 toxic effects included neutropenia (n = 3), thrombocytopenia (n = 5), diarrhea (n = 2), dehydration (n = 2), hypertension (n = 2), fatigue (n = 2), nausea/vomiting (n = 1), decreased ejection fraction (n = 2), and vision changes (n = 2). The dose-limiting toxicity rate was 20% (95% CI 1% to 72%) for pazopanib-pretreated patients and 28% (95% CI 15% to 45%) for non-pretreated patients.

### Analysis of Royal Marsden Hospital prognostic score

We also looked into the RMH scoring system^10^ for sarcoma patients in our pazopanib trials (Table 4). The median albumin level was 4.2 g/dL (normal range 3.5 to 4.7 g/dL), the median lactate dehydrogenase level was 479 U/L (normal range 313 to 618 U/L), and the median number of metastatic sites was 2. The most common metastatic site was the lungs, in 35 patients (80%). Twenty-eight of 44 patients had an RMH score of 0, and the median OS in this group was 58 weeks, which was significantly better than the median OS of 23 weeks in the 16 patients who had an RMH score of 1 to 3 (HR = 3.2, 95% CI = 1.6 to 6.4, p = 0.0020; Fig. 3).

**Table 4:** Distribution of patients by RMH prognostic score in the pazopanib combination studies.

RMH score	Number of patients
0	28
1	12
2	3
3	1

### TCGA Data

**Figure 5:**
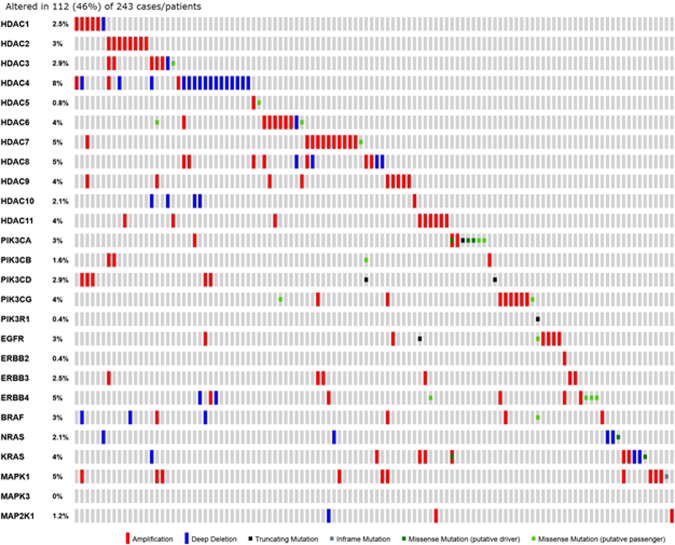
The Cancer Genome Atlas (TCGA) figure was generated to show copy number alterations, and mutations in selected genes namely HDAC, PI3K, HER2, and MAPK/RAS/RAF. The figure panel was created using the cBioPortal [ref: https://www.ncbi.nlm.nih.gov/pubmed/22588877] for the sarcoma data set available on the portal.

Next generation sequencing data from the Cancer Genome Atlas (TCGA) revealed copy number alterations, and mutations in 112/243 (46%) of patients in the HDAC, PI3K, HER2, and MAPK/RAS/RAF pathways (Fig. 5). Specifically HDAC2 and HDAC7 were amplified in 3% and 5% of patients. More patients (41%) had HDAC alterations (HDAC 1–11), compared to patients with MAPK/RAS/RAF (15%) and P13K pathway alterations (12%).

### Assessment of HDAC and co-occuring pathway alterations

In addition we assessed associations between HDAC alterations and other alterations (i.e., is the rate of HDAC alterations higher in patients with the other alterations compared to patients without those alterations).

### MAPK/RAS/RAF pathway

Of the 210 patients without **MAPK/RAS/RAF** alterations, 18% had HDAC alterations while of the 33 patients with **MAPK/RAS/RAF** alterations, 97% had HDAC alterations. Odds Ratio = 144.8 with 95% confidence interval = (19.2, 1093) and p < 0.0001.

### PI3K pathway

Of the 214 patients without PI3K pathway alterations, 27% had HDAC alterations while of the 29 patients with these alterations, 41% had HDAC alterations. Odds Ratio = 1.9 with 95% confidence interval = (0.9, 4.2) and p = 0.11.

### ERBB pathway

Of the 218 patients without ERBB alterations, 27% had HDAC alterations while of the 25 patients with these alterations, 48% had HDAC alterations. Odds Ratio = 2.5 with 95% confidence interval = (1.1, 5.9) and p = 0.025.

We compared marginal proportions of patients with HDAC alterations vs. marginal proportions of patients with other alterations: HDAC: 70/243 = 29%; MAPK/RAS/RAF: 33/243 = 14%; PI3K: 29/243 = 12%; ERBB: 25/243 = 10% HDAC vs MAPK/RAS/RAF: p < 0.0001 HDAC vs PI3K: p < 0.0001; HDAC vs ERBB: p < 0.0001 Note: test = McNemar’s chi-squared test for paired proportions.

## Discussion

We have reported the outcomes of heavily pre-treated bone and soft tissue sarcomas in pazopanib combination studies. The combinations demonstrated safety in patients with refractory soft tissue sarcoma with no unexpected toxicities observed in the combination studies. The soft tissue sarcoma cohort PFS was 14.5 weeks. This is lower than PFS of 4.6 months which was seen with pazopanib in the Phase 3 PALETTE trial. However, the patients in our study were heavily pre-treated and a few had prior VEGF inhibitor therapy. Pazopanib has been approved by the U.S. Food and Drug Administration and the European Medicines Agency for this indication, but its potential is limited by a relatively short PFS advantage and low response rate (9%). It is within this context that we had hypothesized that pazopanib combinations could overcome resistance to monotherapy.

The lack of observed advantage in our study may be due to our small sample size and their heavily pretreated nature. Additionally, nearly half of our sample (41%) consisted of patients with bone sarcomas, which are generally chemotherapy resistant and which have previously been excluded from pazopanib trials^11^. This may explain the reduction inOS and response rates. In our analysis, median PFS for all patients was 9.6 weeks, range 8.4 weeks (in vorinostat cohort) to 18 weeks (in trastuzumab cohort). Interestingly, PFS was higher in patients pretreated with prior pazopanib (n = 5, 15.7 weeks vs 9.6 weeks). ORR was 9% in our analysis. Dose limiting toxicity (DLT) was experienced by 27% of patients (n = 12) in our analysis. DLT rate was noted to be higher in patients who were not pretreated with pazopanib (28% vs 20%).

Prognosis remains poor in metastatic and advanced sarcomas, and few options exist for chemotherapy-refractory disease^12^. Although our study did not show a survival advantage, we did notice an exceptional responder in a patient with pleomorphic sarcoma who had stable disease for 32 weeks. This patient received pazopanib and vorinostat combination therapy. Two other patients, one with a well-differentiated liposarcoma and one with an alveolar soft part sarcoma of the thigh, had long-term stable disease (48.4 and 110.2 weeks, respectively). The first patient received a MEK inhibitor with pazopanib, and the second patient received vorinostat with pazopanib. However, these responses could also be secondary to the natural indolent history of the disease in addition to cytostatic drug effects. We also observed stable disease in 36% of patients, even though most of those cases did not achieve 6 months of stability.

No particular combination showed exceptional efficacy. The vorinostat, everolimus, and MEK inhibitor combinations each led to a partial response in at least one patient and stable disease lasting at least 3 months in multiple patients. Only the vorinostat and MEK inhibitor combinations led to durable stable disease lasting more than 6 months, which may be an early signal of efficacy with these combinations. Because these trials were dose escalation trials performed in unselected sarcoma patients, it would be appropriate to assess clinical benefit of the combinations in patients with sarcoma that have these pathway alterations. One of the critical confounders we recognize in this study is the use of prior TKIs. A relatively large number of patients were pre-treated with and had progressed on another TKI. Therefore, the poor response rate to pazopanib combinations may be a sequelae of TKI failure in general.

None of these trials were biomarker driven. Perhaps selecting patients with respective pathway mutations would improve the response rate of pazopanib combination therapy. We queried TCGA to investigate if a mutation driven approach would be possible. Interestingly, our analysis of the TCGA data revealed that more patients had HDAC alterations (HDAC 1–11), when compared to patient with MAPK/RAS/RAF and P13K pathway alterations. It would be relevant to pursue clinical trials with HDAC inhibitors and other epigenetic pathway targeted therapy in sarcoma. In addition, in depth sequencing and molecular profiling of the exceptional responders in clinical trials would be instructive for future patients especially in rare diseases like sarcoma^13–18^. The strong relationship between HDAC and MAP kinase pathway co-occurring aberration should also be explored.

RMH score^10^ is based on three variables, and the collective score has been shown to be associated with survival in phase 1 trials as well as specifically in sarcomas^19^. We were interested in evaluating the validity of this score in sarcoma patients on tyrosine kinase inhibitors.Our analysis revealed that OS was significantly higher in patients with RMH score of 0 compared with those with RMH score of 1 to 3 (58 weeks vs 23 weeks). The RMH appears to be valid when applied to patients on pazopanib. Our findings validate and are consistent with those of a prior report in bone sarcomas patients enrolled in phase 1 trials^20^ and soft tissue sarcomas^21^.

Taking our small sample size into account, it does not appear that patients pretreated with pazopanib derived benefit from retreatment with combination therapy. Whether they had primary resistance to pazopanib or the combination was unable to overcome acquired resistance is unknown. The sample size was insufficient to evaluate patients based on prior response to TKIs. We feel that these patients would be better served on clinical trials with medications not including pazopanib or other anti-angiogenic agents. In addition the dose of pazopanib is crucial as well^22,23^ and some of these patients may have received lower dosing in escalation cohorts. We acknowledge that the pazopanib dose is a major limitation of this study. The nature of dose escalation phase 1 trials means that some early patients on the trial may get a sub-therapeutic dose. The group of patients that received pazopanib less than 400 mg daily most likely had a sub-therapeutic dose and this may have contributed to the poor overall response rate in our study.

Our study did not identify unexpected toxicities for any pazopanib combinations with inhibitors of the HDAC, mTOR, or MEK pathways. We found partial responses in four patients (9%) and prolonged stable disease >6 months in another four patients (9%). This preliminary evidence supports further evaluation of the pazopanib combinations in sarcoma. Given that our data from TCGA shows a larger proportion of patients with HDAC pathway (HDAC1–11) alterations further evaluation of epigenetic pathway inhibitor trials are warranted.
